# Rural–urban differences in out‐of‐network treatment initiation and engagement rates for substance use disorders

**DOI:** 10.1111/1475-6773.14299

**Published:** 2024-03-08

**Authors:** Eli Raver, Sheldon M. Retchin, Yiting Li, Andrew D. Carlo, Wendy Y. Xu

**Affiliations:** ^1^ Division of Health Services Management and Policy, College of Public Health The Ohio State University Columbus Ohio USA; ^2^ Division of General Internal Medicine, Department of Internal Medicine, College of Medicine The Ohio State University Columbus Ohio USA; ^3^ Meadows Mental Health Policy Institute Northwestern University Feinberg School of Medicine Chicago Illinois USA; ^4^ Present address: Nationwide Children's Hospital Columbus Ohio USA

**Keywords:** alcohol‐related disorders, managed care programs, opioid‐related disorders, provider networks, rural health services, substance‐related disorders

## Abstract

**Objective:**

To examine rural–urban disparities in substance use disorder treatment access and continuation.

**Data Sources and Study Setting:**

We analyzed a 2016–2018 U.S. national secondary dataset of commercial insurance claims.

**Study Design:**

This cross‐sectional study examined individuals with a new episode of opioid, alcohol, or other drug use disorders. Treatment initiation and engagement rates, and rates of using out‐of‐network providers for these services, were compared between rural and urban patients.

**Data Collection:**

We included individuals 18–64 years old with continuous employer‐sponsored insurance.

**Principal Findings:**

Patients in rural settings experienced lower treatment initiation rates for alcohol (36.6% vs. 38.0%, *p* < 0.001), opioid (41.2% vs. 44.2%, *p* < 0.001), and other drug (37.7% vs. 40.1%, *p* < 0.001) use disorders, relative to those in urban areas. Similarly, rural patients had lower treatment engagement rates for alcohol (15.1% vs. 17.3%, *p* < 0.001), opioid (21.0% vs. 22.6%, *p* < 0.001), and other drug (15.5% vs. 17.5%, *p* < 0.001) use disorders. Rural patients had higher out‐of‐network rates for treatment initiation for other drug use disorders (20.4% vs. 17.2%, *p* < 0.001), and for treatment engagement for alcohol (27.6% vs. 25.2%, *p* = 0.006) and other drug (36.1% vs. 31.1%, *p* < 0.001) use disorders.

**Conclusions:**

These findings indicate that individuals with substance use disorders in rural areas have lower rates of initial and ongoing treatment, and are more likely to seek care out‐of‐network.

**What is known on this topic**

- Treatment for substance use disorders is underutilized, even for those with commercial health insurance.
- Rural areas have additional barriers to accessing care, including limited networks of healthcare providers who contract with commercial insurance.

**What this study adds**

- Individuals with substance use disorders in rural areas receive initial and ongoing treatment less often than their urban counterparts.
- For patients that do receive treatment, those in rural areas are more likely to go outside their insurance network for treatment of alcohol and other (non‐opioid) use disorders.

## 1 INTRODUCTION

Nearly 35 million working‐age Americans report having a substance use disorder (SUD) involving alcohol, opioids, or other drugs.^1^, ^2^ Nationwide, drug overdose deaths nearly doubled from 2015 to 2020 and remain a leading cause of death.^1^, ^3^ Although rural and urban areas experience similar rates of SUD, rural areas have experienced faster growth in substance‐related deaths over the last two decades.^1^, ^4^, ^5^ Timely initiation of SUD treatment and engagement in ongoing treatment can reduce morbidity and mortality and improve social outcomes.^6^, ^7^

There are particular concerns over access to effective and ongoing care in rural areas, which suffer from higher rates of alcohol, prescription opioid, and methamphetamine use disorders than urban areas.^1^, ^8^ Successful treatment for these conditions often involves sustained engagement with both behavioral counseling and medication.^9^, ^10^, ^11^, ^12^ Because these treatments rely on a sufficient workforce of providers trained to treat SUDs, provider shortages in rural areas may be especially pertinent to accessing ongoing SUD treatment.^13^, ^14^, ^15^

As of 2020, private insurance covered 58% of non‐elderly adults with SUD, primarily through employer‐sponsored managed care plans with a defined network of providers.^2^ However, psychiatrists are less likely than other physician specialties to participate in insurance networks.^16^ Seeing out‐of‐network (OON) providers typically means much higher out‐of‐pocket costs and longer travel times. While enrollees can choose to see OON providers, there have also been policy concerns that inadequate provider networks can create substantial financial burdens for adults with SUD.^17^ In rural areas, where long travel distances for in‐person visits and provider shortages already present barriers to care,^13^ inadequate provider networks may compound barriers to accessing timely and continued SUD treatment.

Yet, there has been no empirical evaluation of the rural disparities in SUD treatment among privately insured individuals, and how OON care utilization differs between rural and urban areas. Therefore, this study aims to examine disparities in access to SUD treatments and continuation of treatments by comparing commercially insured rural and urban enrollees with SUD for: rates of initial and ongoing treatment for SUD; and rates of initial and ongoing treatment for SUD from OON providers.

## 2 METHOD

### 2.1 Data and study sample

This cross‐sectional study pooled 2016–2018 data from the IBM MarketScan® Commercial Claims and Encounters Database. This nationwide health insurance claims database includes detailed information about diagnosis, treatments, medications, care setting, date of service, provider network status, plan types, and rudimentary demographic data for approximately 40 million individuals annually,^18^ corresponding to the U.S. population of approximately 155 million individuals covered by employer‐sponsored insurance annually during the study period.^19^ The COVID‐19 pandemic had made accessing reliable healthcare utilization data after 2019 very challenging. Regardless, rural barriers to accessing care and heterogeneous provider network standards persist today. Therefore, the study findings using earlier data still provide important evidence on OON SUD treatment. The Ohio State University Institutional Review Board exempted this study from review because it used de‐identified data.

We examined new episodes of SUD for individuals aged 18 to 64 years who were continuously enrolled in employer‐sponsored insurance during the study period. Based on Healthcare Effectiveness Data and Information Set (HEDIS) measures to identify alcohol or other drug use disorders in claims data,^20^ we defined a new episode of SUD as an outpatient or inpatient encounter associated with a diagnosis of SUD, based on ICD‐10 codes, and no prior history of SUD for at least 60 days before the index episode.^20^ Following this definition, individuals could have multiple new episodes of SUD throughout the study period. Our study included three separate samples for episodes of alcohol use disorder, opioid use disorder, and other drug use disorders, which included cannabis, sedatives/hypnotics, cocaine, other stimulants, hallucinogens, inhalants, and other psychoactive substances.

### 2.2 Outcome measures

We employed four outcome measures. Using the HEDIS algorithm for Initiation and Engagement of Alcohol and Other Drug Abuse or Dependence Treatment,^20^ we determined two outcome measures of SUD treatment utilization: “initiation”, which captured whether individuals initiated treatment within 14 days of a new episode of SUD, and “engagement”, which measured whether individuals engaged in two or more SUD treatment encounters within 34 days after initiation. Treatments included inpatient admissions, outpatient visits, telehealth encounters, and medications. Treatment engagement is conditional on treatment initiation, but the denominator for both outcomes included all new episodes of SUD to remain consistent with HEDIS measures.^20^ Thus, the initiation rate is an upper bound for the engagement rate.

To measure the proportionate utilization of SUD treatments from OON providers, we created two binary measures: whether patients utilized OON services for treatment initiation, and whether patients utilized OON services for at least one treatment engagement encounter. The denominators for each OON service utilization outcome were patients who had treatment initiation and engagement, respectively. Thus, the OON treatment rates represent the proportion of SUD episodes with treatment initiation that involved OON services, and the proportion of SUD episodes with treatment engagement that involved OON services.

### 2.3 Statistical analysis

Our key variable of analysis was rurality, defined as residence outside of a metropolitan statistical area, following delineations by the U.S. Office of Management and Budget.^21^ We used logistic regression models to estimate the likelihood of treatment initiation and engagement, and the likelihood of utilizing OON services for treatment initiation and engagement, between rural and urban patients. The logistic regression models controlled for age, sex, census division, health plan type (grouped by typical provider network structure),^18^ and Charlson Comorbidity Index score.^22^

Based on the regression model estimates, we calculated the treatment rates and OON service utilization rates as average adjusted predictions using true covariate values, for rural and urban patients. Statistical differences between rural and urban groups were captured by the average marginal effects.

Robust standard errors clustered by patients accounted for the possibility of multiple episodes per patient. All *p*‐values are from two‐sided tests, with statistical significance set at α = 0.05.

## 3 RESULTS

As shown in Table 1, the samples included 206,137 new episodes of alcohol use disorder, 67,718 new episodes of opioid use disorder, and 117,927 new episodes of other drug use disorders. Rural enrollees accounted for 9.6% of alcohol use disorder episodes, 12.9% of opioid use disorder episodes, and 9.7% of new episodes of other drug use disorders. Among new episodes of any type of SUD, rural enrollees were more likely to be enrolled in preferred provider organization (PPO) plans and less likely to be enrolled in health maintenance organization (HMO) plans, compared to urban enrollees.

**TABLE 1:** Demographic, comorbidity, and health plan data for new episodes of substance use disorders among enrollees in employer‐sponsored insurance plans.

Sample characteristics	Alcohol use disorder (*N* = 206,137)		Opioid use disorder (*N* = 67,718)		Other drug use disorders (*N* = 117,927)	
	Rural	Urban	Rural	Urban	Rural	Urban
New episodes, *n* (%)	19,729 (9.6)	186,408 (90.4)	8709 (12.9)	59,009 (87.1)	11,396 (9.7)	106,531 (90.3)
Female, *n* (%)	6263 (31.7)	68,329 (36.7)	4371 (50.2)	27,789 (47.1)	4787 (42.0)	41,708 (39.2)
Age in years, mean (SD)	42.9 (14.3)	40.9 (14.1)	41.7 (14.2)	40.8 (14.2)	34.0 (14.1)	33.0 (13.6)
Charlson Comorbidity Index score, mean (SD)	0.81 (1.55)	0.72 (1.48)	0.86 (1.61)	0.90 (1.67)	0.57 (1.28)	0.58 (1.33)
Health plan type, *n* (%)						
Comprehensive	1100 (5.6)	10,461 (5.6)	518 (6.0)	4113 (7.0)	614 (5.4)	6801 (6.4)
HMO, EPO	1411 (7.2)	20,914 (11.2)	564 (6.5)	5975 (10.1)	759 (6.7)	12,749 (12.0)
PPO, POS	11,850 (60.1)	102,006 (54.7)	5538 (63.6)	33,990 (57.6)	7015 (61.6)	58,212 (54.7)
HDHP, CDHP	5368 (27.2)	53,027 (28.5)	2089 (24.0)	14,931 (25.3)	3008 (26.4)	28,769 (27.0)

Abbreviations: CDHP, consumer‐directed health plan; EPO, exclusive provider organization; HDHP, high deductible health plan; HMO, health maintenance organization; POS, point of service; PPO, preferred provider organization.

Treatment initiation and engagement were low in all SUD groups. Treatment initiation rates were 37.8% for alcohol use disorder, 43.8% for opioid use disorder, and 39.8% for other drug use disorders. Treatment engagement rates were 17.1% for alcohol use disorder, 22.4% for opioid use disorder, and 17.3% for other drug use disorders.

Overall, regression‐adjusted results revealed that rural patients experienced slightly lower treatment initiation and engagement rates for each type of SUD studied. As shown in Table 2, new episodes among rural patients had lower treatment initiation rates than among urban patients for alcohol use disorder (36.6% vs. 38.0%, *p* < 0.001), opioid use disorder (41.2% vs. 44.2%, *p* < 0.001), and other drug use disorders (37.7% vs. 40.1%, *p* < 0.001). New episodes of SUD among rural patients had lower treatment engagement rates compared to those among urban patients for alcohol use disorder (15.1% vs. 17.3%, *p* < 0.001), opioid use disorder (21.0% vs. 22.6%, *p* = 0.001), and other drug use disorders (15.5% vs. 17.5%, *p* < 0.001).

**TABLE 2:** Adjusted rural and urban rates of treatment initiation and engagement for new episodes of alcohol, opioid, and other drug use disorders.

	Adjusted rate, %^a^			*p*‐value^b^
	Overall	Rural	Urban	
Treatment initiation				
Alcohol use disorder (*n* = 206,137)	37.8	36.6	38.0	<0.001
Opioid use disorder (*n* = 67,718)	43.8	41.2	44.2	<0.001
Other drug use disorders (*n* = 117,927)	39.8	37.7	40.1	<0.001
Treatment engagement				
Alcohol use disorder (*n* = 206,137)	17.1	15.1	17.3	<0.001
Opioid use disorder (*n* = 67,718)	22.4	21.0	22.6	<0.001
Other drug use disorders (*n* = 117,927)	17.3	15.5	17.5	<0.001

^a^ The adjusted rates are the average adjusted predictions from logistic regression models, using true covariate values. The regression models controlled for age, sex, Census division, health plan type, and comorbid health conditions.

^b^ *p*‐values are for the average marginal effects of rural versus urban settings.

Whereas treatment rates were consistently lower in rural settings, the rural–urban differences in OON utilization varied by SUD group. As shown in Table 3, new episodes of SUD among rural patients had higher OON utilization for treatment initiation of other drug use disorders (20.4% vs. 17.2%, *p* < 0.001), compared to those among urban patients. However, differences in OON utilization for treatment initiation of alcohol use disorder (14.0% vs. 13.2%, rural vs. urban, *p* = 0.06) and opioid use disorder (16.9% vs. 18.0%, rural vs. urban, *p* = 0.10) were nominal and failed to reach statistical significance. For new episodes of SUD that received treatment engagement, rural patients had significantly higher OON utilization for alcohol use disorder (27.6% vs. 25.2%, *p* = 0.006) and other drug use disorders (36.1% vs. 31.1%, *p* < 0.001) compared to those among urban patients. Nonetheless, there were no significant rural–urban differences in the OON utilization of treatment engagement for opioid use disorder (34.1% vs. 34.0%, *p* = 0.93).

**TABLE 3:** Adjusted rural and urban rates of out‐of‐network service utilization for treatment initiation and engagement for new episodes of alcohol, opioid, and other drug use disorders.

	Adjusted rate, %^a^			*p*‐value^b^
	Overall	Rural	Urban	
Out‐of‐network treatment initiation				
Alcohol use disorder (*n* = 78,002)	13.2	14.0	13.2	0.06
Opioid use disorder (*n* = 29,647)	17.9	16.9	18.0	0.10
Other drug use disorders (*n* = 46,965)	17.5	20.4	17.2	<0.001
Out‐of‐network treatment engagement				
Alcohol use disorder (*n* = 35,236)	25.4	27.6	25.2	0.006
Opioid use disorder (*n* = 15,170)	34.0	34.1	34.0	0.93
Other drug use disorders (*n* = 20,440)	31.5	36.1	31.1	<0.001

^a^ The adjusted rates are the average adjusted predictions from logistic regression models, using true covariate values. The regression models controlled for age, sex, Census division, health plan type, and comorbid health conditions. The out‐of‐network treatment rates represent the proportion of substance use disorder episodes with treatment initiation that involved out‐of‐network services, and the proportion of substance use disorder episodes with treatment engagement that involved out‐of‐network services.

^b^ *p*‐values are for the average marginal effects of rural versus urban settings.

## 4 DISCUSSION

Rural patients with SUD have traditionally faced more barriers compared with similar urban‐dwelling patients for accessing timely and continued treatment, such as fewer specialists who can provide medication or behavioral therapy, longer travel times to access care, and social stigma.^13^, ^15^ Our study found that rural patients with new episodes of SUD had significantly lower treatment rates than similar patients in urban areas. Patients in rural areas receiving SUD treatment also experienced rates of OON care that were at least as large as patients in urban areas, depending on the type of SUD. While the observed rural disparities were small, any differences are concerning given that overall treatment rates were low and that treatment often involved out‐of‐network providers.

Our findings are consistent with previous studies documenting underutilization of SUD treatment,^1^, ^23^ and more pronounced barriers to care in rural areas.^13^, ^14^, ^15^ While one in five Americans live in rural areas, the population density is insufficient to sustain specialized SUD treatment centers or specialist providers. Although primary care providers have increasingly taken on screening for and treating SUD, patients in isolated rural areas or with long travel distances are the least likely to receive follow‐up care for SUD.^24^ This is especially concerning because sustained treatment engagement is critical for treatment success, including improved health and employment outcomes.^10^, ^11^ While there are overall low rates of SUD treatment engagement, rural patients appear to be particularly vulnerable to barriers to sustained SUD treatment.

Our findings of high rates of OON utilization for SUD treatment are supported by prior evidence that health plan provider networks have limited capacity to care for patients with behavioral health conditions.^17^, ^25^, ^26^ Although the Affordable Care Act requires private health plan networks to provide sufficient access to behavioral health care, “sufficiency” is not defined. Accordingly, most state regulations on network adequacy lack explicit standards for behavioral health generally, or SUD management specifically.^27^, ^28^ Because of the limited behavioral health workforce in rural areas, primary care providers, including nurse practitioners and physician assistants, often meet the demand for SUD treatment within provider networks.^24^, ^29^ Despite the Mental Health Parity and Addiction Equity Act of 2008 and the Health Care and Education Reconciliation Act of 2010, which prevent health insurers from providing less favorable benefits for mental health and SUD than for physical health, patients continue to face disproportionate cost‐sharing burden when seeking OON treatment for SUD.^30^, ^31^ For example, PPO plans offer OON coverage at non‐preferred rates, and were more prevalent among rural enrollees with SUD. Higher rates of OON care could indicate inadequate provider networks in rural areas, which impose additional costs and travel burdens on rural patients.^17^, ^32^

We observed heterogeneous findings for OON treatment of different types of SUD, which speaks to the nuances of addressing rural–urban disparities in network access. For instance, there were no disparities observed for OON treatment of opioid use disorders. Much of the policy efforts to address SUD during the last two decades have focused on the opioid epidemic.^33^, ^34^ This may have balanced rural and urban network access to treatment for opioid use disorder while leaving disparities unaddressed for other SUD types. Additionally, while telehealth can improve rural access to SUD treatment,^35^ it may be most beneficial for opioid use disorder, which has more effective medication options available through remote prescribing.^9^

Addressing rural–urban disparities in SUD treatment rates and OON utilization will likely require a combination of approaches. One possible solution is to train a new cohort of rural providers to treat SUD as it is experienced by rural patients. This could include focusing on the types of SUD more common in rural areas, such as alcohol and methamphetamine use disorders, or addressing practical issues more common in rural areas, such as prescription opioid diversion and fentanyl use.^8^, ^29^ This approach takes time to increase provider expertise and does not necessarily increase the workforce capacity. Meanwhile, it may be necessary to relax geographic proximity standards for provider networks in areas experiencing shortages of behavioral health providers. Allowing more distant specialty providers and facilities to be in‐network may require some patients to travel farther for care, but it would mitigate the increased cost‐sharing that otherwise accompanies OON or non‐preferred provider visits. This is especially important, as higher cost‐sharing can delay initiating treatment or interrupt ongoing treatment.^36^ Additionally, as telehealth becomes increasingly prevalent in rural areas,^37^ geographic proximity may become less important for network adequacy requirements. In particular, the Collaborative Care Model allows primary care providers working with a behavioral healthcare manager to treat SUD in collaboration with a psychiatric consultant, typically via telehealth, while coordinating billing through the primary care practice.^38^, ^39^ For situations where a large proportion of patients already resort to OON care, such as rural treatment of SUD, relaxed geographic proximity requirements for provider networks could be a net benefit to these vulnerable patients.

### 4.1 Limitations

Our study had several notable limitations. Although the HEDIS algorithms are validated standards, the claims data may miss patients who did not have accurate diagnostic coding. Additionally, even for commercially insured enrollees, we were unable to observe services that were paid entirely out‐of‐pocket. This could produce lower estimates of treatment rates and OON service utilization. However, there is no reason to suspect that biased estimates of treatment rates were associated with rural or urban enrollees. Although SUD treatment rates may differ by race and ethnicity,^1^ this information was not present in the claims data, and thus could not be included in the regression models. The definition of rurality was limited to the level of metropolitan statistical area where patients resided. Since this definition may categorize some rural areas as metropolitan,^21^ the observed rural–urban differences may be biased toward the null. Nonetheless, other researchers have used this definition of rural and urban areas with MarketScan® claims data.^37^, ^40^

This study focused on commercial insurance, which represents only 16% of SUD spending,^41^ and may not be generalizable to patients who are uninsured or who are covered by Medicare or Medicaid. These individuals are overrepresented in rural areas,^42^ and may be less likely to access SUD treatment due to limited resources available or fewer providers who accept their insurance.^43^, ^44^, ^45^ Thus, the rural–urban disparities in SUD treatment rates and OON service utilization are likely to be even greater for the general population than the estimates we observed in this study.

## 5 CONCLUSIONS

While there are low treatment rates for all patients with SUD, rural patients are less likely than their urban counterparts to receive timely initial and ongoing treatment for SUDs. Further, heterogeneous rural–urban differences in the use of OON services for treatment of alcohol, opioid, and other drug use disorders suggest important nuances, and even trade‐offs, in managing different SUDs in rural settings. Policymakers should consider the potential for differential impacts of any efforts to address network access to treatment for SUD, both between rural and urban areas and between different types of SUD.

## CONFLICT OF INTEREST STATEMENT

Dr. Retchin reports fees and stock options from Aveanna Healthcare as an independent director, outside the submitted work. Dr. Carlo reports consulting fees from Otsuka Pharmaceutical, and honoraria from the Mid‐America Mental Health Technology Transfer Center, outside the submitted work. Drs. Raver, Li, and Xu have nothing to disclose.

## Supporting information


**Data S1.** Supplementary Information.
