# Supplementary material for: Rural–urban differences in out‐of‐network treatment initiation and engagement rates for substance use disorders
Source: Health Serv Res. 2024 Mar 8;59(5):e14299. doi: 10.1111/1475-6773.14299 (PMC11366955; doi:10.1111/1475-6773.14299)
Supplement: Supplementary file 1 — Data S1. Supplementary Information. [file HESR-59-0-s001.docx]

**SUPPLEMENTAL APPENDIX**

Rural-Urban Differences in Out-of-Network Treatment Initiation and Engagement Rates for Substance Use Disorders

**Table S1** Logistic regression model of treatment initiation following new episodes of alcohol, opioid, and other drug use disorders, by enrollee characteristics

|  | Alcohol use disorder^a^  (n=206,137) | Opioid use disorder^b^  (n=67,718) | Other drug use disorders^c^  (n=117,927) |
| --- | --- | --- | --- |
|  | Average Marginal Effect (Standard Error) | | |
| Urban | [Reference] | [Reference] | [Reference] |
| Rural | -0.014***  (0.004) | -0.029***  (0.006) | -0.023***  (0.005) |
| Male | [Reference] | [Reference] | [Reference] |
| Female | -0.019***  (0.002) | -0.072***  (0.004) | -0.037***  (0.003) |
| Age 18-24 | [Reference] | [Reference] | [Reference] |
| Age 25-34 | 0.034***  (0.004) | -0.116***  (0.006) | -0.043***  (0.004) |
| Age 35-44 | 0.043***  (0.004) | -0.262***  (0.006) | -0.070***  (0.004) |
| Age 45-54 | 0.031***  (0.003) | -0.363***  (0.006) | -0.086***  (0.005) |
| Age 55-64 | -0.014***  (0.003) | -0.429***  (0.006) | -0.151***  (0.005) |
| Charlson Comorbidity Index | 0.025***  (0.001) | 0.011***  (0.001) | 0.012***  (0.001) |
| Comprehensive Health Plan | [Reference] | [Reference] | [Reference] |
| HMO,^d^ EPO^e^ | -0.047***  (0.006) | -0.066***  (0.010) | -0.081***  (0.008) |
| PPO,^f^ POS^g^ | -0.021***  (0.005) | -0.041***  (0.008) | -0.031***  (0.007) |
| HDHP,^h^ CDHP^i^ | -0.040***  (0.005) | -0.045***  (0.009) | -0.048***  (0.007) |
| New England | [Reference] | [Reference] | [Reference] |
| Mid-Atlantic | 0.036***  (0.005) | -0.002  (0.011) | 0.107***  (0.008) |
| East North Central | 0.029***  (0.005) | -0.032**  (0.010) | 0.059***  (0.007) |
| West North Central | 0.039***  (0.006) | -0.079***  (0.013) | 0.052***  (0.008) |
| South Atlantic | 0.030***  (0.005) | -0.050***  (0.010) | 0.034***  (0.007) |
| East South Central | 0.061***  (0.006) | -0.121***  (0.011) | 0.048***  (0.008) |
| West South Central | 0.058***  (0.006) | -0.147***  (0.011) | 0.069***  (0.008) |
| Mountain | 0.027***  (0.006) | -0.078***  (0.012) | 0.031***  (0.009) |
| Pacific | 0.030***  (0.005) | -0.031**  (0.011) | 0.057***  (0.008) |

^a^Logistic regression results are based on 206,137 observations of new episodes of alcohol use disorder. Robust standard errors were adjusted for 179,281 clusters, with each cluster being an enrollee who could have more than one new episode during the study period. The model Wald chi-squared statistic with 18 degrees of freedom was 1780.15. Coefficients for reference groups are 0.

^b^Logistic regression results are based on 67,718 observations of new episodes of opioid use disorder. Robust standard errors were adjusted for 57,475 clusters, with each cluster being an enrollee who could have more than one new episode during the study period. The model Wald chi-squared statistic with 18 degrees of freedom was 7186.88. Coefficients for reference groups are 0.

^c^Logistic regression results are based on 117,927 observations of new episodes of other drug use disorders. Robust standard errors were adjusted for 104,405 clusters, with each cluster being an enrollee who could have more than one new episode during the study period. The model Wald chi-squared statistic with 18 degrees of freedom was 1694.41. Coefficients for reference groups are 0.

^d^HMO, health maintenance organization

^e^EPO, exclusive provider organization

^f^PPO, preferred provider organization

^g^POS, point of service

^h^HDHP, high deductible health plan

^i^CDHP, consumer directed health plan

*p<0.05; **p<0.01; ***p<0.001

**Table S2** Logistic regression model of treatment engagement following new episodes of alcohol, opioid, and other drug use disorders, by enrollee characteristics

|  | Alcohol use disorder^a^  (n=206,137) | Opioid use disorder^b^  (n=67,718) | Other drug use disorders^c^  (n=117,927) |
| --- | --- | --- | --- |
|  | Average Marginal Effect (Standard Error) | | |
| Urban | [Reference] | [Reference] | [Reference] |
| Rural | -0.022***  (0.003) | -0.016***  (0.005) | -0.020***  (0.004) |
| Male | [Reference] | [Reference] | [Reference] |
| Female | -0.011***  (0.002) | -0.063***  (0.003) | -0.043***  (0.002) |
| Age 18-24 | [Reference] | [Reference] | [Reference] |
| Age 25-34 | 0.024***  (0.003) | -0.074***  (0.006) | -0.015***  (0.003) |
| Age 35-44 | 0.025***  (0.003) | -0.177***  (0.006) | -0.028***  (0.003) |
| Age 45-54 | 0.006*  (0.003) | -0.256***  (0.005) | -0.045***  (0.004) |
| Age 55-64 | -0.035***  (0.003) | -0.302***  (0.005) | -0.088***  (0.004) |
| Charlson Comorbidity Index | 0.004***  (0.001) | -0.010***  (0.001) | -0.003**  (0.001) |
| Comprehensive Health Plan | [Reference] | [Reference] | [Reference] |
| HMO,^d^ EPO^e^ | -0.023***  (0.005) | -0.054***  (0.008) | -0.043***  (0.006) |
| PPO,^f^ POS^g^ | 0.003  (0.004) | -0.015*  (0.007) | 0.003  (0.005) |
| HDHP,^h^ CDHP^i^ | -0.015***  (0.004) | -0.027***  (0.007) | -0.013*  (0.005) |
| New England | [Reference] | [Reference] | [Reference] |
| Mid-Atlantic | 0.022***  (0.004) | -0.013  (0.009) | 0.068***  (0.006) |
| East North Central | 0.006  (0.004) | -0.036***  (0.009) | 0.019***  (0.005) |
| West North Central | 0.004  (0.005) | -0.063***  (0.011) | 0.032***  (0.006) |
| South Atlantic | -0.006  (0.004) | -0.047***  (0.009) | 0.015**  (0.005) |
| East South Central | 0.001  (0.005) | -0.093***  (0.009) | 0.012*  (0.006) |
| West South Central | 0.008  (0.004) | -0.092***  (0.009) | 0.032***  (0.006) |
| Mountain | -0.015***  (0.005) | -0.084***  (0.010) | 0.002  (0.006) |
| Pacific | 0.021***  (0.004) | -0.051***  (0.009) | 0.045***  (0.006) |

^a^Logistic regression results are based on 206,137 observations of new episodes of alcohol use disorder. Robust standard errors were adjusted for 179,281 clusters, with each cluster being an enrollee who could have more than one new episode during the study period. The model Wald chi-squared statistic with 18 degrees of freedom was 1037.64. Coefficients for reference groups are 0.

^b^Logistic regression results are based on 67,718 observations of new episodes of opioid use disorder. Robust standard errors were adjusted for 57,475 clusters, with each cluster being an enrollee who could have more than one new episode during the study period. The model Wald chi-squared statistic with 18 degrees of freedom was 6044.94. Coefficients for reference groups are 0.

^c^Logistic regression results are based on 117,927 observations of new episodes of other drug use disorders. Robust standard errors were adjusted for 104,405 clusters, with each cluster being an enrollee who could have more than one new episode during the study period. The model Wald chi-squared statistic with 18 degrees of freedom was 1607.50. Coefficients for reference groups are 0.

^d^HMO, health maintenance organization

^e^EPO, exclusive provider organization

^f^PPO, preferred provider organization

^g^POS, point of service

^h^HDHP, high deductible health plan

^i^CDHP, consumer directed health plan

*p<0.05; **p<0.01; ***p<0.001

**Table S3** Logistic regression model of using out-of-network services for treatment initiation following new episodes of alcohol, opioid, and other drug use disorders, by enrollee characteristics

|  | Alcohol use disorder^a^  (n=78,002) | Opioid use disorder^b^  (n=29,647) | Other drug use disorders^c^  (n=46,965) |
| --- | --- | --- | --- |
|  | Average Marginal Effect (Standard Error) | | |
| Urban | [Reference] | [Reference] | [Reference] |
| Rural | 0.008  (0.004) | -0.011  (0.007) | 0.032***  (0.007) |
| Male | [Reference] | [Reference] | [Reference] |
| Female | -0.001  (0.003) | -0.015***  (0.005) | -0.014***  (0.004) |
| Age 18-24 | [Reference] | [Reference] | [Reference] |
| Age 25-34 | -0.057***  (0.005) | -0.071***  (0.006) | -0.003  (0.005) |
| Age 35-44 | -0.082***  (0.004) | -0.121***  (0.007) | -0.022***  (0.005) |
| Age 45-54 | -0.098***  (0.004) | -0.123***  (0.007) | -0.028***  (0.006) |
| Age 55-64 | -0.115***  (0.004) | -0.140***  (0.007) | -0.047***  (0.007) |
| Charlson Comorbidity Index | -0.008***  (0.001) | -0.007***  (0.002) | -0.003  (0.002) |
| Comprehensive Health Plan | [Reference] | [Reference] | [Reference] |
| HMO,^d^ EPO^e^ | -0.063***  (0.006) | -0.090***  (0.010) | -0.077***  (0.008) |
| PPO,^f^ POS^g^ | 0.038***  (0.006) | 0.056***  (0.009) | 0.064***  (0.007) |
| HDHP,^h^ CDHP^i^ | 0.041***  (0.006) | 0.069***  (0.010) | 0.070***  (0.008) |
| New England | [Reference] | [Reference] | [Reference] |
| Mid-Atlantic | 0.034***  (0.006) | 0.043***  (0.011) | 0.037***  (0.009) |
| East North Central | -0.024***  (0.005) | 0.005  (0.010) | -0.028**  (0.009) |
| West North Central | -0.039***  (0.006) | -0.015  (0.013) | -0.041***  (0.010) |
| South Atlantic | 0.027***  (0.006) | 0.042***  (0.010) | 0.028**  (0.009) |
| East South Central | 0.014*  (0.007) | 0.012  (0.012) | -0.004  (0.010) |
| West South Central | 0.016*  (0.006) | 0.027*  (0.012) | 0.010  (0.010) |
| Mountain | 0.046***  (0.007) | 0.068***  (0.013) | 0.072***  (0.011) |
| Pacific | 0.042***  (0.006) | 0.039***  (0.011) | 0.058***  (0.010) |

^a^Logistic regression results are based on 78,002 observations of new episodes of alcohol use disorder that received treatment initiation. Robust standard errors were adjusted for 72,275 clusters, with each cluster being an enrollee who could have more than one new episode during the study period. The model Wald chi-squared statistic with 18 degrees of freedom was 2242.26. Coefficients for reference groups are 0.

^b^Logistic regression results are based on 29,647 observations of new episodes of opioid use disorder that received treatment initiation. Robust standard errors were adjusted for 26,924 clusters, with each cluster being an enrollee who could have more than one new episode during the study period. The model Wald chi-squared statistic with 18 degrees of freedom was 1104.47. Coefficients for reference groups are 0.

^c^Logistic regression results are based on 46,965 observations of new episodes of other use disorders that received treatment initiation. Robust standard errors were adjusted for 43,837 clusters, with each cluster being an enrollee who could have more than one new episode during the study period. The model Wald chi-squared statistic with 18 degrees of freedom was 994.05. Coefficients for reference groups are 0.

^d^HMO, health maintenance organization

^e^EPO, exclusive provider organization

^f^PPO, preferred provider organization

^g^POS, point of service

^h^HDHP, high deductible health plan

^i^CDHP, consumer directed health plan

*p<0.05; **p<0.01; ***p<0.001

**Table S4** Logistic regression model of using out-of-network services for treatment engagement following new episodes of alcohol, opioid, and other drug use disorders, by enrollee characteristics

|  | Alcohol use disorder^a^  (n=35,236) | Opioid use disorder^b^  (n=15,170) | Other drug use disorders^c^  (n=20,440) |
| --- | --- | --- | --- |
|  | Average Marginal Effect (Standard Error) | | |
| Urban | [Reference] | [Reference] | [Reference] |
| Rural | 0.024**  (0.009) | 0.001  (0.012) | 0.051***  (0.012) |
| Male | [Reference] | [Reference] | [Reference] |
| Female | 0.011*  (0.005) | -0.011  (0.008) | 0.002  (0.007) |
| Age 18-24 | [Reference] | [Reference] | [Reference] |
| Age 25-34 | -0.090***  (0.008) | -0.112***  (0.010) | -0.024**  (0.009) |
| Age 35-44 | -0.127***  (0.008) | -0.193***  (0.011) | -0.075***  (0.010) |
| Age 45-54 | -0.123***  (0.007) | -0.192***  (0.012) | -0.074***  (0.011) |
| Age 55-64 | -0.152***  (0.008) | -0.224***  (0.014) | -0.113***  (0.013) |
| Charlson Comorbidity Index | -0.008***  (0.002) | 0.000  (0.003) | 0.007*  (0.003) |
| Comprehensive Health Plan | [Reference] | [Reference] | [Reference] |
| HMO,^d^ EPO^e^ | -0.087***  (0.011) | -0.122***  (0.018) | -0.110***  (0.015) |
| PPO,^f^ POS^g^ | 0.103***  (0.010) | 0.128***  (0.016) | 0.124***  (0.014) |
| HDHP,^h^ CDHP^i^ | 0.129***  (0.011) | 0.164***  (0.017) | 0.156***  (0.015) |
| New England | [Reference] | [Reference] | [Reference] |
| Mid-Atlantic | 0.042***  (0.010) | 0.088***  (0.018) | 0.048**  (0.017) |
| East North Central | -0.029**  (0.010) | 0.013  (0.017) | -0.027  (0.016) |
| West North Central | -0.062***  (0.011) | -0.010  (0.022) | -0.057**  (0.018) |
| South Atlantic | 0.100***  (0.010) | 0.088***  (0.017) | 0.103***  (0.017) |
| East South Central | 0.036**  (0.013) | 0.033  (0.020) | 0.010  (0.019) |
| West South Central | 0.057***  (0.012) | 0.055**  (0.020) | 0.057**  (0.018) |
| Mountain | 0.110***  (0.013) | 0.148***  (0.022) | 0.179***  (0.021) |
| Pacific | 0.061***  (0.011) | 0.090***  (0.019) | 0.105***  (0.018) |

^a^Logistic regression results are based on 35,236 observations of new episodes of alcohol use disorder that received treatment engagement. Robust standard errors were adjusted for 33,633 clusters, with each cluster being an enrollee who could have more than one new episode during the study period. The model Wald chi-squared statistic with 18 degrees of freedom was 1694.13. Coefficients for reference groups are 0.

^b^Logistic regression results are based on 15,170 observations of new episodes of opioid use disorder that received treatment engagement. Robust standard errors were adjusted for 14,208 clusters, with each cluster being an enrollee who could have more than one new episode during the study period. The model Wald chi-squared statistic with 18 degrees of freedom was 945.31. Coefficients for reference groups are 0.

^c^Logistic regression results are based on 20,440 observations of new episodes of other drug use disorders that received treatment engagement. Robust standard errors were adjusted for 19,502 clusters, with each cluster being an enrollee who could have more than one new episode during the study period. The model Wald chi-squared statistic with 18 degrees of freedom was 943.46. Coefficients for reference groups are 0.

^d^HMO, health maintenance organization

^e^EPO, exclusive provider organization

^f^PPO, preferred provider organization

^g^POS, point of service

^h^HDHP, high deductible health plan

^i^CDHP, consumer directed health plan

*p<0.05; **p<0.01; ***p<0.001
